# SSeCKS/AKAP12 scaffolding functions suppress B16F10-induced peritoneal metastasis by attenuating CXCL9/10 secretion by resident fibroblasts

**DOI:** 10.18632/oncotarget.20092

**Published:** 2017-08-09

**Authors:** Masashi Muramatsu, Lingqiu Gao, Jennifer Peresie, Benjamin Balderman, Shin Akakura, Irwin H. Gelman

**Affiliations:** ^1^ Institute of Resource Development and Analysis, Kumamoto University, Kumamoto 860-0811, Japan; ^2^ Department of Cancer Genetics, Roswell Park Cancer Institute, Buffalo 14263, NY, USA; ^3^ Frontiers in Bioscience Research Institute in Aging and Cancer, Irvine 92618, CA, USA

**Keywords:** SSeCKS/AKAP12, metastasis, senescent secretome, CXCL9/10, CXCR3

## Abstract

SSeCKS/Gravin/AKAP12 (SSeCKS) is a kinase scaffolding protein known to suppress metastasis by attenuating tumor-intrinsic PKC- and Src-mediated signaling pathways [1]. In addition to downregulation in metastatic cells, *in silico* analyses identified SSeCKS downregulation in prostate or breast cancer-derived stroma, suggesting a microenvironmental cell role in controlling malignancy. Although orthotopic B16F10 and SM1WT1[*Braf*^V600E^] mouse melanoma tumors grew similarly in syngeneic WT or SSeCKS-null (KO) mice, KO hosts exhibited 5- to 10-fold higher levels of peritoneal metastasis, and this enhancement could be adoptively transferred by pre-injecting naïve WT mice with peritoneal fluid (PF), but not non-adherent peritoneal cells (PC), from naïve KO mice. B16F10 and SM1WT1 cells showed increased chemotaxis to KO-PF compared to WT-PF, corresponding to increased PF levels of multiple inflammatory mediators, including the Cxcr3 ligands, Cxcl9 and 10. *Cxcr3* knockdown abrogated enhanced chemotaxis to KO-PF and peritoneal metastasis in KO hosts. Conditioned media from KO peritoneal membrane fibroblasts (PMF), but not from KO-PC, induced increased B16F10 chemotaxis over controls, which could be blocked with Cxcl10 neutralizing antibody. KO-PMF exhibited increased levels of the senescence markers, SA-β-galactosidase, p21^waf1^ and p16^ink4a^, and enhanced Cxcl10 secretion induced by inflammatory mediators, lipopolysaccharide, TNFα, IFNα and IFNγ. SSeCKS scaffolding-site mutants and small molecule kinase inhibitors were used to show that the loss of SSeCKS-regulated PKC, PKA and PI3K/Akt pathways are responsible for the enhanced Cxcl10 secretion. These data mark the first description of a role for stromal SSeCKS/AKAP12 in suppressing metastasis, specifically by attenuating signaling pathways that promote secretion of tumor chemoattractants in the peritoneum.

## INTRODUCTION

The vast majority of patients with carcinomas succumb to metastatic disease, yet most current therapies target cell proliferation or survival mechanisms employed by primary tumor cells. For a macroscopic metastasis to form, tumor cells must transiently adopt various cell biologies, motilities and signaling pathways, such as those governing epithelial to mesenchymal to amoeboid bi-directional transitions, in order to transverse from primary tumor sites, through the blood or lymphatic systems, and ultimately to distal sites of colonization and growth [2–4]. There is growing appreciation for the role of the tumor microenvironment (TME), including stromal, vascular and immune cells, in the formation of the so-called metastatic niche (MN) [5], envisioned by Paget as the “soil” for the tumor cell “seed” [6]. Specifically, TME cells secrete multiple proteins that facilitate metastatic niche formation through the recruitment of and crosstalk with tumor and bone marrow-derived stem cell populations [7]. Recent data suggest that tumor cells remotely activate pre-metastatic niche (PMN) sites via the transfer of mRNAs and miRNAs contained within exosomes that are shed into the blood stream [8–10], as well as by TME altering factors, such as IL-4, IL-6, CSF-1 and VEGF, secreted by tumors [11]. Current thinking suggests that tumor cells help create distal PMN sites in this manner, producing micro-foci of inflammation in which TME cells are induced to secrete chemoattractants such as SDF-1, EGF and multiple chemokines [7]. Dissection of TME-tumor crosstalk pathways [7, 12, 13] has identified mechanisms controlling chemoattraction of tumor and bone marrow stem cells to PMN sites, adherence and survival of trafficking cells to these sites (colonization), tumor cell dormancy vs. active proliferation, metastatic neovascularization, and drug resistance. Layered on top of this mechanism is a further concept that aging (i.e., increased cellular senescence) abets this process through the increased, chronic secretion by TME cells of inflammatory mediators [14, 15]. This has led to efforts to antagonize metastatic growth by therapeutically targeting TME-mediated inflammation in the MN [16, 17].

SSeCKS/Gravin/AKAP12 is among a growing list of so-called metastasis suppressors, defined as gene products that can experimentally suppress specific parameters of metastatic progression while having limited or no role in primary tumor formation [18]. SSeCKS was first described as being transcriptionally downregulated by oncogenically activated Src [19, 20], and consistent with the notion that increasing Src-family kinase (SFK) activation levels plays a critical role in metastasis formation [18, 21, 22], SSeCKS is downregulated in many metastases compared to primary tumors in the same organ type [1, 23]. SSeCKS deficiency is used as a predictive marker of tumor aggressiveness in colon, gastric, esophageal and prostate cancer [1]. For example, Mardin et al. [24] showed that AKAP12 downregulation due to promoter hypermethylation correlates with increased clinical rates of metastasis and invasion. In addition, compared to primary-site lesions, roughly a third of prostate cancer metastases show chromosomal loss of the *AKAP12* locus in 6q24-25.2 [1, 25]. We showed that SSeCKS/AKAP12 loss correlates with a more rapid onset of clinical post-castration metastasis compared to cases with no loss (5.4 vs. 15 months, respectively) [26]. Consistent with its suggested role as a metastasis suppressor, the loss of SSeCKS in transgenic (Tg) mice with prostate-specific *Rb* deficiency induces lymph node metastases even though only high grade intraepithelial neoplasia form in the prostates [26]. Additionally, compared to WT mice, SSeCKS-null Tg mice are metastasis-prone in a DMBA/TPA-induced skin carcinogenesis model [27]. Interestingly, SSeCKS-null mice exhibit epidermal hyperplasia marked by an upregulation of FAK, a known promoter of skin carcinogenesis [28].

The loss of SSeCKS may also promote metastasis by resulting in premature cell senescence. For example, SSeCKS-deficient Tg mice, though physiologically normal, exhibit hyperplasias in organs typically enriched for SSeCKS expression, such as the prostate [29]. SSeCKS-null prostates also express markers of increased senescence, such as senescence-associated β-galactosidase (SA-β-gal), p16^Ink4a^ and γH2AX [30]. Indeed, SSeCKS-null mouse embryo fibroblasts (MEF) suffer from an Rb-dependent senescence, and are marked by a senescence-associated secretory phenotype (SASP) that includes VEGF and IL-6 [30].

The major mechanism by which SSeCKS is thought to manifest its metastasis-suppressing activity in tumor cells is through its ability to scaffold key signaling mediators in a spatiotemporal manner [1], partly facilitated by SSeCKS containing binding domains for plasma membrane sites as well as for F-actin [31, 32]. For example, regulation of premature senescence is controlled by SSeCKS scaffolding of PKCα and δ isoforms [30], whereas regulation of chemotaxis, invasiveness and cell adhesion are controlled by scaffolding domains for PKC, Src and plasma membrane binding sites [33–35]; regulation of G1→S transition is controlled by scaffolding domains for cyclins [36].

The forced re-expression of SSeCKS reversed parameters of *Src*- and *Ras*-induced oncogenic transformation in fibroblasts or epithelial cells *in vitro*, including anchorage- and growth factor-independence, the loss of contact inhibition, actin stress fibers and mature focal adhesion plaques, and the gain of chemotaxis activity and invasiveness [23, 34, 35], the latter involving suppression of Rho family-dependent invadopodia (podosome) formation [37]. In contrast, SSeCKS re-expression had little effect on *s.c.* tumor formation or growth, or on the colonization rate of metastatic tumors cells in the lung, yet this caused a severe decrease in the formation of lung macrometastases [34, 38], correlating with the downregulation of HIF-1α-mediated VEGF expression. Indeed, the forced VEGF expression in these cells partially rescued formation of macrometastases [39].

The ability of SSeCKS to regulate neovascularization in the MN at the tumor level parallels that of SFK in regulating this process through their expression in TME cells. For example, Weis et al. [40] showed that in Src- or Yes-null (vs. WT) hosts, *i.v.* tumor cell inoculation resulted in avascular pulmonary micrometastases due to interrupted VEGFR2→SFK→VE-cadherin signaling in vascular endothelial cells that suppressed their recruitment to the MN. This suggests that in regards to MN formation, the yin-yang relationship between SSeCKS and Src may control multiple crosstalk pathways between tumor and ME cells.

CXCR3 is a receptor for a subset of chemokines that lack the so-called glutamic acid-leucine-arginine (ELR) motif, including CXCL9/MIG, CXCL10/IP10, CXCL11/ITAC/IP9 and CXCL4/PF4. Upregulated CXCR3 expression in human breast, melanoma, renal and colon tumors correlates with poor prognosis [41, 42]. Although the tumor-specific expression of CXCR3 ligands, such as CXCL10, can induce tumor suppression by recruiting T- and NK-cells [43], many studies have shown that increased tumor cell expression of CXCR3 correlates with increased metastatic potential owing to an increased chemotactic response to ligands expressed by PMN cells [44–50]. Indeed, high CXCL10 expression in the PMN correlates with poor outcomes in melanoma, colon and renal cancers [51].

Based on the ability of SSeCKS/AKAP12 to attenuate Src-mediated metastatic signaling at the tumor cell level, and based on roles for TME-expressed SFK in regulating metastasis (above), we addressed whether SSeCKS also plays a role in the TME to regulate metastatic potential. SSeCKS is widely expressed in fibroblasts and smooth muscle, in select epithelial and endothelial cells, and in specialized cells such as Purkinje, mesangial and parietal cells in the renal glomerulus, and in pericytes [52]. Here we show that the loss of SSeCKS in the TME results in a metastasis-prone environment in the peritoneum for syngeneic melanoma tumor cells, without affecting the growth rate of orthotopic tumors. This is likely mediated through a CXCR3-dependent chemoattraction of tumor cells to the enhanced secretion of the chemokine ligands, CXCL9 and 10, by peritoneal membrane fibroblasts (PMF). Our data indicate that SSeCKS normally suppresses this pathway in PMF through the direct scaffolding of PKC, PKA or activators of PI3K/Akt signaling.

## RESULTS

**Figure 1:**
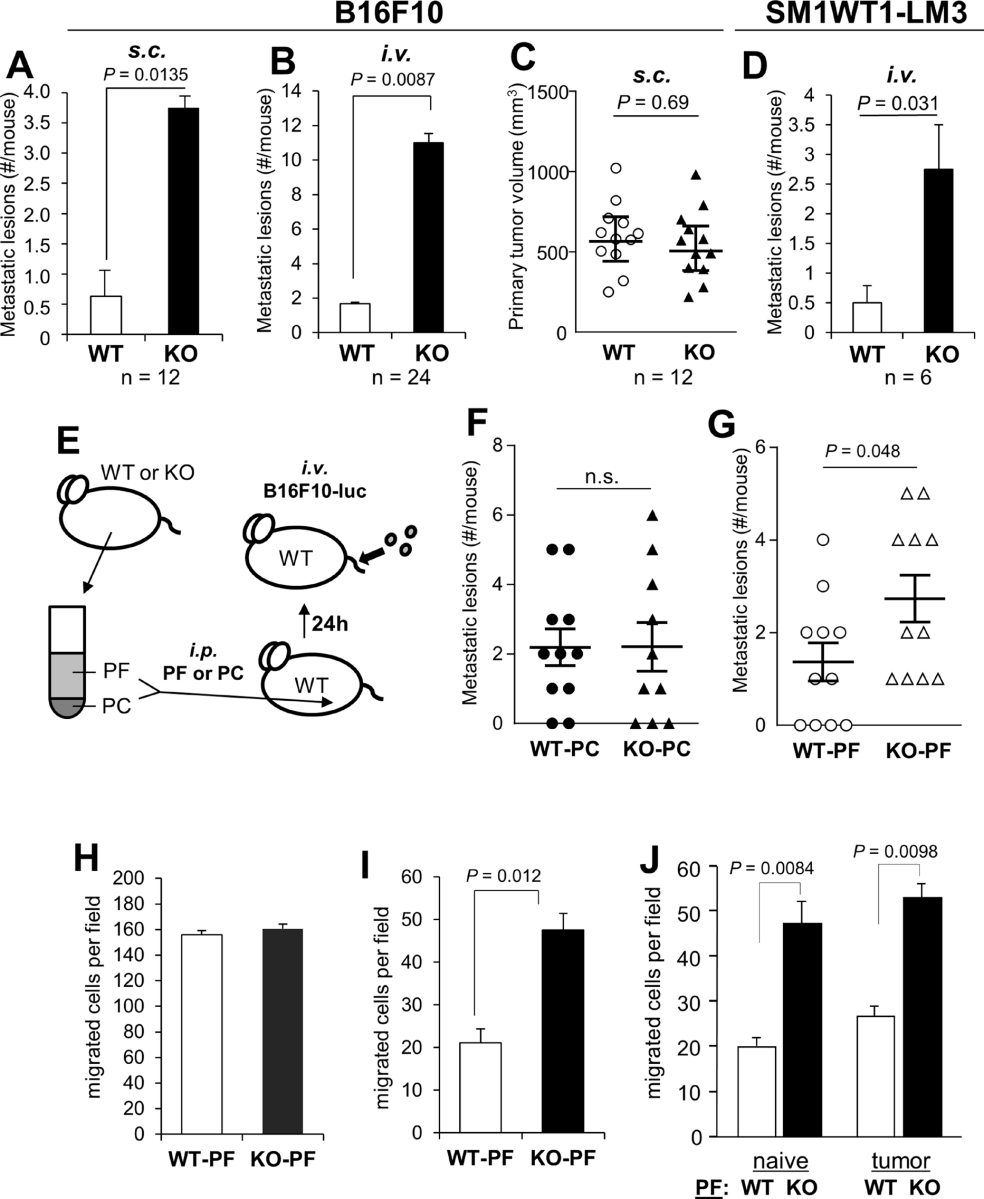
SSeCKS-null (KO) mice exhibit increased potential for peritoneal metastasis **(A)** The percent of WT vs. KO mice exhibiting macroscopic peritoneal metastases after *s.c.* injection of B16F10 cells. **(B)** The average number of macrometastases/mouse after *i.v.* injection of B16F10-luc. **(C)** Tumor volumes induced by B16F10-luc injected *s.c.* in WT vs. KO mice. **(D)** The average number of macrometastases/mouse after *i.v.* injection of SM1WT1-LM3-luc. **(E)** Schematic of adoptive transfer of either peritoneal fluid (PF) or peritoneal cells (PC) from naïve WT or KO mice to WT hosts (via *i.p.* injection), followed the next day by tail vein *i.v.* injection of B16F10-luc cells. **(F)** Number of metastatic lesions/mouse following the adoptive transfer of WT- or KO-PC. **(G)** Number of metastatic lesions/mouse following the adoptive transfer of WT- or KO-PF. **(H)** Migration of B16F10-luc in wound-healing assays containing 20% PF from WT- or KO-mice. The number of cells migrating into 6 microscopic fields were quantified and averaged. **(I)** Chemotaxis assays (through Boyden chambers) by B16F10-luc cells towards media (no serum) containing 20% PF from WT- or KO-mice (fluid flushed and pooled from 3 mice each, normalized for total protein content). **(J)** Similar chemotaxis assay as in panel H except using PF from naïve or tumored (*s.c.*) WT- or KO-mice.

There is a growing appreciation for the notion that metastasis-regulating genes, such as *KISS1, BRMS1, NME1* or *SMAD4*, exert their effects at both the tumor and TME level [53]. Indeed, there is a corpus of data showing that SSeCKS/AKAP12 is especially downregulated in prostate, breast, colon, gastric and pancreatic metastases compared to primary tumors and normal tissues, and that SSeCKS re-expression suppresses parameters of metastatic growth but has little effect on primary-site tumor growth [1, 26]. Preliminary analysis of Oncomine data indicates that SSeCKS/AKAP12 levels are also downregulated in the tumor-associated stroma of prostate and breast cancer relative to stroma in non-neoplastic control tissue [54–56] (Supplementary Figure 1). We addressed whether TME-encoded SSeCKS might play a role in metastatic potential by injecting WT or KO mice with syngeneic B16F10 melanoma cells *i.v.* or at orthotopic sites (*s.c.*). Roughly 44% of patients with metastatic melanoma have peritoneal lesions [57], and although B16F10 cells do not encode mutated *Braf* (equivalent to the BRAF^V600E^ allele found in roughly 40–50% of human melanoma), they have equivalent mutations in the three tumor suppressors, *Pten*,*Cdkn2a*, and *Trp53* [58] found in human melanomas with WT-BRAF [59, 60]. KO mice uniformly exhibited a 7–10 fold increase in the numbers of macroscopic peritoneal metastases compared to WT hosts whether the tumor cells were injected orthotopically (Figure 1A) or *i.v.* (Figure 1B; Supplementary Figure 2A). In contrast, there was no statistical difference in the growth of *s.c.* primary-site tumors (Figure 1C). Interestingly, the *i.v.* injection of B16F10 cells also resulted in increased metastatic burden in the lungs and liver of KO mice (Supplementary Figure 2C). In contrast, metastasis from orthotopic sites seemed to favor the peritoneum and to a lesser extent, the lung, but not the kidney, liver or spleen (Supplementary Figure 2D). This suggests that the loss of SSeCKS in peritoneal cells renders this “soil” environment metastasis-prone.

To rule out the possibility that the small percentage of strain 129 alleles remaining in our KO mice (after 7 back-crosses into the C57BL/6 background) might selectively promote increased metastatic potential, we produced *Akap12*^+/+^ and *Akap12*^−/−^ mice from *Akap12*^+/−^ stocks, all of which contain the same level of contaminating 129 background, and then injected B16F10 cells stably expressing luciferase (“B16F10-luc”) cells *i.v.* via tail-veins. Again, KO mice exhibited roughly a 9-fold increase in the formation of macrometastases compared to the genetically equivalent WT mice (Supplementary Figure 2B), strongly arguing that the increased metastatic potential derived only from the loss of SSeCKS in the TME.

We then addressed whether a *Braf*-driven melanoma would also show increased peritoneal metastasis in KO hosts. First, we produced a metastatic variant (LM3) of SM1WT1 [61], a transplantable melanoma from a C57BL/6 transgenic line bearing a *Braf*^*T1799A*^ mutation that encodes the V600E variant found in human melanomas, by passaging lung tumors (3 rounds) produced by *i.v.* injection of male C57BL/6J mice. Parallel injection of WT vs. KO mice with SM1WT1-LM3 produced roughly 5-fold higher peritoneal metastases in KO hosts (Figure 1D).

**Figure 2:**
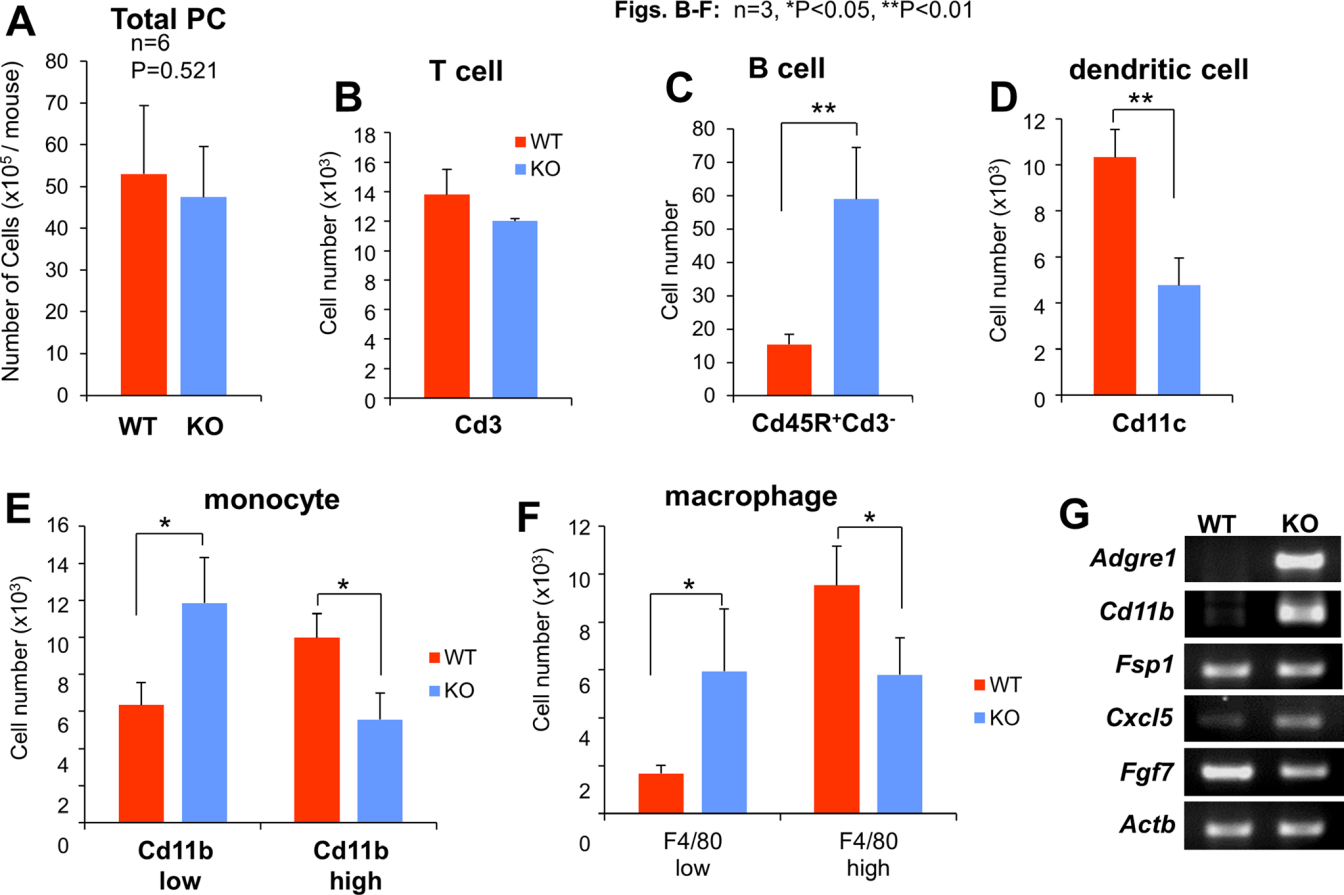
Profile of peritoneal cell (PC) subgroups based on cytometric analyses Although the average total number of PC/mouse or T-lymphocytes (Cd3^+^) did not differ between WT and KO mice (**A** and **B**), KO mice had increased numbers of B-lymphocytes (Cd45R^+^Cd3^−^) and immature monocytes and macrophages (Cd11b^low^ and F4/80^low^, respectively), whereas WT mice had higher levels of dendritic cells (Cd11c^+^) and mature monocytes and macrophages (Cd11b^hi^ and F4/80^hi^, respectively) (**B**–**F**). (**G**) Semi-quantitative RT-PCR for mouse *Adgre1* (F: 5′-GGATGTACAGATGGGGGATG, R: 5′-GGAAGCCTCGTTTACAGGTG), *Cd11b* (F: AAACCACAGTCCCGCAGAGA, R: CGTGTTCACCAGCTGGCTTA), *Fsp1* (F: TGAGCAACTTGGACAGCAACA, R: TTCCGGGGTTCCTTATCTGGG), *Cxcl5* (F: GCATTTCTGTTGCTGTTCACGCTG, R: CCTCCTTCTGGTTTTTCAGTTTAGC), *Fgf7* (F: TTTGGAAAGAGCGACGACTT, R: GGCAGGATCCGTGTCAGTAT) and *Actb* (F: ACCTTCTACAATGAGCTGCG, R: CTGGATGGCTACGTACATGG) from WT- or KO-PMF RNA.

In order to determine whether the enhanced metastatic potential in KO mice could be adoptively transferred by either cell-free peritoneal fluid (PF) or non-adherent cells in the peritoneal cavity (PC), i.e.- the two major components of the metastatic “soil”, we performed peritoneal flushes using PBS on tumor-naïve KO or WT mice, then separated PF from PC by centrifugation. WT mice were then injected *i.p.* with PF or PC, and then injected *i.v.* the next day with B16F10-luc cells (Figure 1E). We noted that although the total number of PC/ml or T-cells did not vary between WT and KO mice (Figure 2A, 2B), KO-PC contained higher relative numbers of B-cells and lower numbers of dendritic cells (Figure 2C, 2D). Interestingly, KO-PC contained fewer mature and more immature monocytes and macrophages (based on expression levels of F4/80 and Cd11b markers, respectively) (Figure 2E, 2F). Whereas the transfer of PC from WT or KO mice did not affect B16F10-luc metastasis rates in WT hosts (Figure 1F), transfer of KO-PF enhanced metastasis-forming activity over WT-PF (Figure 1G).

The notion that KO-PF contains increased levels of tumor chemoattractants is consistent with our finding that soluble factors encode metastasis-enhancing activity. To test this notion, we subjected B16F10-luc cells to cell motility assays using WT- or KO-PF as conditioned media (CM) supplements. The ability of tumor cells to migrate in monolayer wound-healing assays was not affected by WT- or KO-PF (Figure 1H), whereas chemotaxis in Boyden chambers was enhanced 2.0- to 2.6-fold by KO-PF (Figure 1I, 1J), but not further enhanced by KO-PF isolated from tumored (*s.c.*) mice (Figure 1J). Thus, it is likely that KO mice secrete higher levels of chemoattractants into their peritoneal cavities compared to WT mice.

**Figure 3:**
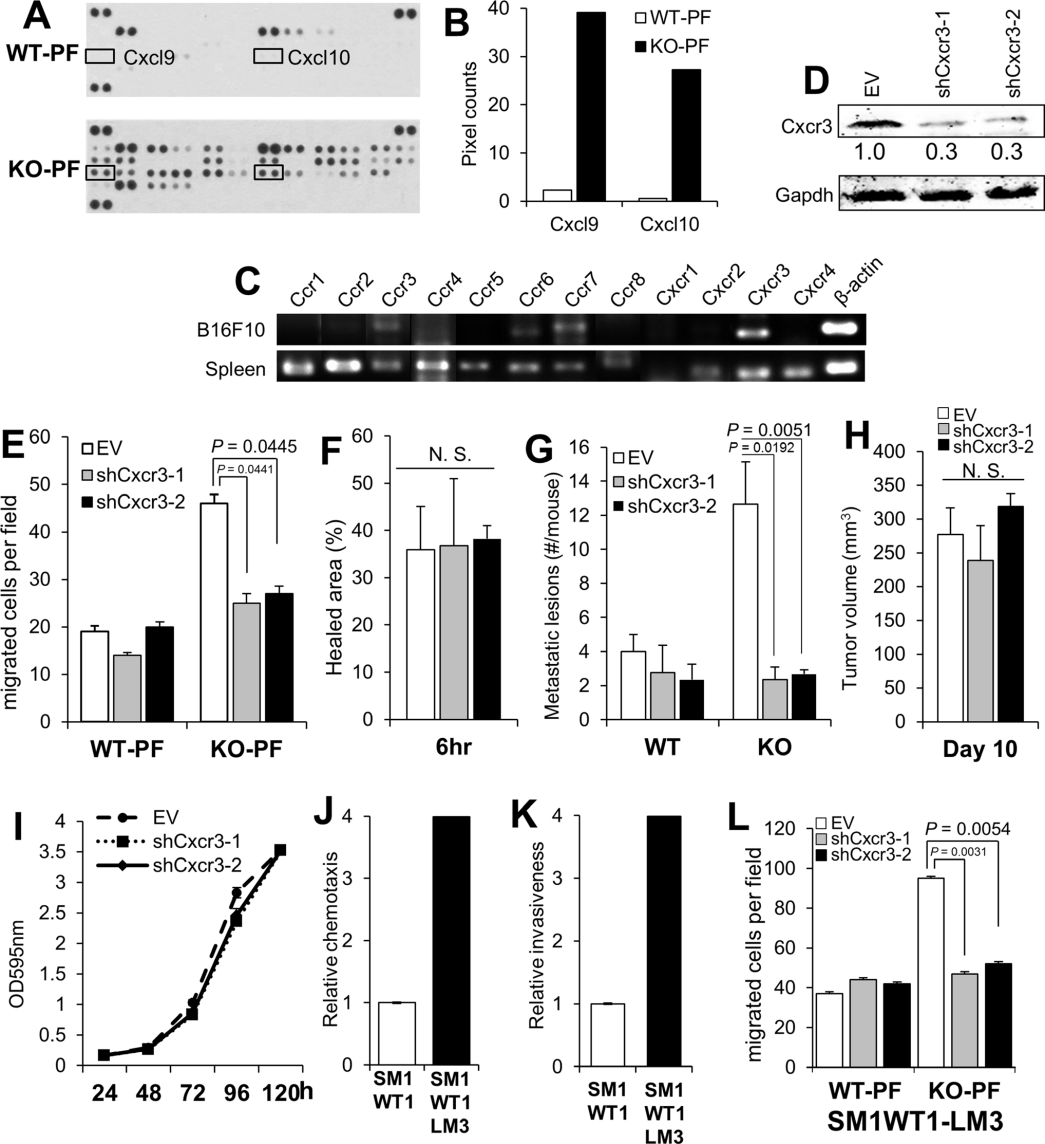
Melanoma-encoded Cxcr3 is required for the enhanced peritoneal metastasis in KO mice and for the enhanced chemotaxis to KO-PF (**A**) Antibody array blots of equal protein aliquots of PF from WT- or KO-mice, identifying the paired spots for Cxcl9 and 10 (boxes). (**B**) Quantification of the pixel counts in the blot in panel A. (**C**) Semi-quantitative RT-PCR for various chemokine receptors in B16F10 cells, using total spleen cells from WT mice as a control. The primer sets used are described previously [62]. (**D**) IB of B16F10-luc stably expressing empty vector (EV) or clones of Cxcr3-specific shRNA, blotted for either Cxcr3 or Gapdh. (**E**) Chemotaxis assay of B16F10-luc cells shown in panel 2D towards media containing 20% PF from WT- or KO-mice. (**F**) Percent healed area of wound monolayers from the cells in panel E in media containing 20% KO-PF. (**G**) Number of peritoneal metastases/mouse after the *i.v.* injection of B16F10-luc cells (EV, shCxcr3-1 or shCxcr3-2) into WT- or KO hosts (*n* = 12 each). (**H**) Tumor volumes (*s.c.*) of B16F10-luc[EV], B16F10-luc[shCxcr3-1] or B16F10-luc[shCxcr3-2] cells in KO mice (*n* = 6). (**I**) Relative cell numbers of B16F10-luc[EV], B16F10-luc[shCxcr3-1] or B16F10-luc[shCxcr3-2] cells. (**J**) Relative chemotaxis of SM1WT1-luc or SM1WT1-LM3-luc cells towards media containing 10% FBS. *P* < 0.0001. (**K**) Relative Matrigel invasiveness of SM1WT1-luc or SM1WT1-LM3-luc cells. *P* < 0.0001. (**L**) Chemotaxis assay towards media containing 20% PF from WT- or KO-mice by SM1WT1-LM3-luc cells transduced with EV, shCxcr3-1 or -2.

In order to identify the potential chemoattractants responsible for the enhanced metastasis-forming activity in KO mice, equal total protein levels of PF from WT or KO mice were applied to antibody arrays specific for chemokines, cytokines and interleukins (R&D Proteome Profiler Mouse Chemokine Array). In contrast to complement C5a and soluble ICAM-1, whose levels did not change between WT- and KO-PF, KO-PF exhibited increased levels of many interleukins, cytokines- such as IFNγ, G-CSF and TNFα, and chemokines (Supplementary Figure 3A). In particular, two Cxcr3 ligands, Cxcl9 and 10, were elevated > 15-fold in the KO-PF array over WT-PF controls (Figure 3A, 3B). It is noteworthy that C57BL/6 mice lack another Cxcr3 ligand, Cxcl11, due to a frame-shift error (National Center for Biotechnology Information Mouse Genomic Sequence Database, accession nos. NT_109320, NW_001030791, NT_039339). Indeed, of the major chemokine receptors, Cxcr3 RNA is expressed highest in B16F10 (Figure 3C) when compared to mouse spleen RNA, which shows uniformly high expression of the major receptor families. Moreover, Cxcr3 expression is upregulated 12-fold in the more metastatic F10 variant of B16 melanoma cells compared to the poorly metastatic F1 variant [62].

To determine if B16F10-expressed Cxcr3 is required for the enhanced metastasis-forming activity in KO mice, B6F10-luc cell lines with stable knockdown of Cxcr3 were developed (Figure 3D) and subjected to migration and metastasis assays. The loss of Cxcr3 ablated the enhanced chemotactic activity of tumor cells towards KO-PF (Figure 3E) whereas Cxcr3 loss had no significant effect on wound-healing migration (Figure 3F). The loss of Cxcr3 also ablated the enhanced peritoneal metastasis in KO vs. WT hosts (Figure 3G; Supplementary Figure 3B), but had no significant effect on the ability to grow as *s.c.* orthotopic tumors in WT hosts (Figure 3H). Moreover, Cxcr3 loss did not affect *in vitro* 2D proliferation of B16F10 cells (Figure 3I). Similarly, SM1WT1-LM3 cells, which displayed increased chemotaxis (Figure 3J) and Matrigel invasiveness (Figure 3K) compared to parental SM1WT1 cells, exhibited increased chemotaxis to KO-PF in a Cxcr3-dependent manner (Figure 3L) but no change in 2D proliferation (Supplementary Figure 3C). Taken together, these data strongly suggest that KO peritonea secrete high levels of Cxcr3 ligands, thereby facilitating increased melanoma chemoattraction in KO mice.

**Figure 4:**
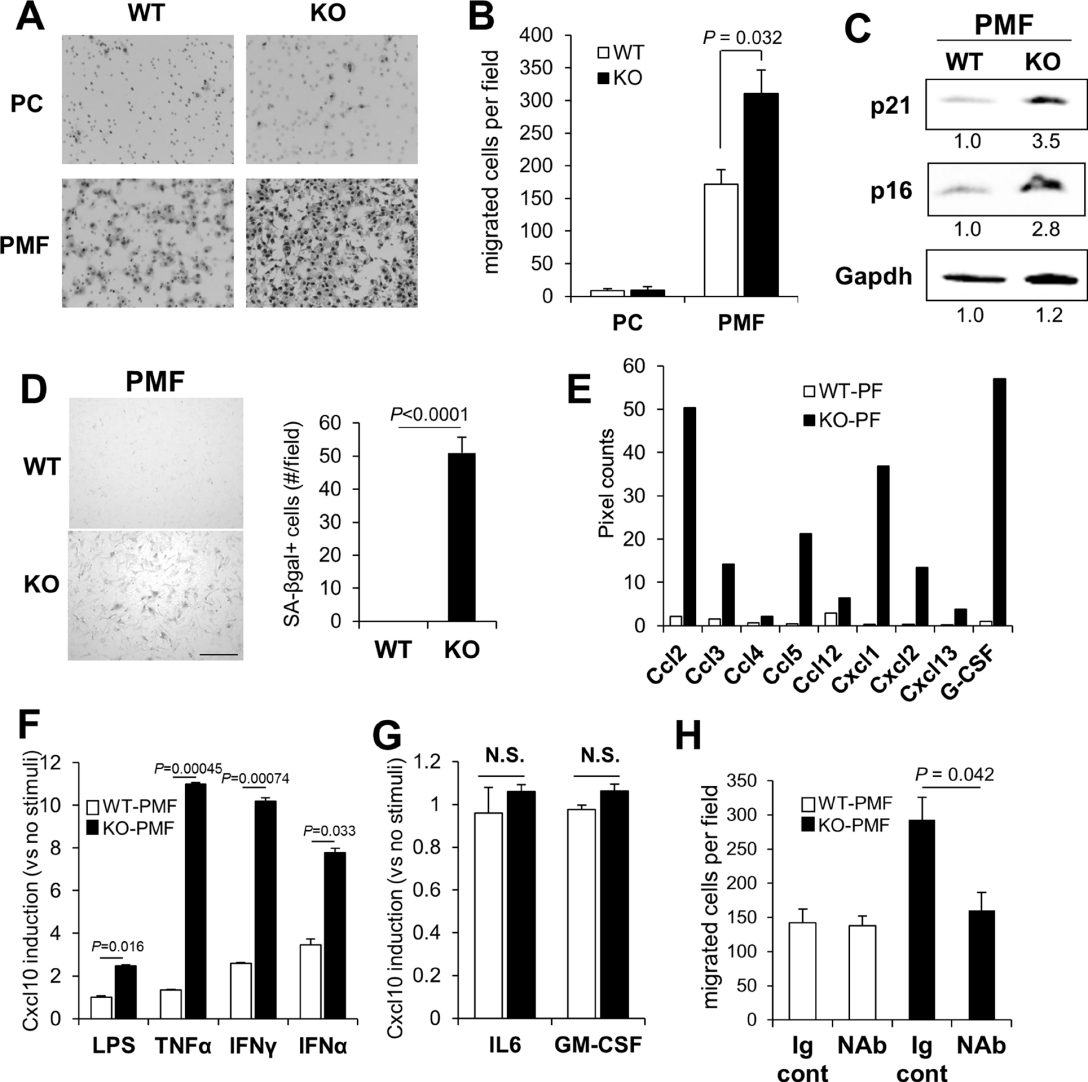
KO-peritoneal membrane fibroblasts (PMF) express increased Cxcl10 levels B16F10-luc migration towards conditioned media from WT- or KO-PC or -PMF (**A** and **B**). (**C**) IB of WT- or KO-PMF probed for p21, p16 or Gapdh (the latter as a protein loading control). Relative pixel counts/lane, normalized to WT-PMF (numbers underneath), were quantified using GeneTools software on a Chemi-Genius^2^ bioimaging system (Syngene, Frederick, MD). (**D**) Staining of WT- or KO-PMF for SA-βgal (left panel), quantified by counting 100 cells in 6 independent fields (right panel). Bar, 100 µm. (**E**) Relative pixel counts for chemokines and G-CSF in the Proteome Profiler Array mouse chemokine array analysis shown in Figure 2A. (**F**) Relative Cxcl10 production in serum-starved WT- vs. KO-PMF treated with LPS, TNFα, IFNγ or IFNα for 30 min. (**G**) Relative CXCL10 production in serum-starved WT- vs. KO-PMF treated with IL6 or GM-CSF for 30 min. N.S., not significant. (**H**) Chemotaxis of B16F10-luc cells to containing conditioned media from WT- or KO-PMF pre-incubated with Ig control or Cxcl10 NAb.

Cxcl9 and 10 are typically produced by myeloid-lineage cells involved in inflammatory responses, such as bone marrow derived monocyte/macrophages [63], however, they are also known to be secreted by mesenchymal cells in inflammatory niches [64, 65] or due to senescence [66–69]. Thus, we determined whether CM from KO-PC, which have relatively lower levels of Cd11b-marked myeloid cells (Figure 2E), or from PMF, adherent cells that are a major component of peritoneal membranes, could produce increased levels of melanoma chemoattractants. PMF likely derive from resident membrane fibroblasts as well as from mesothelial cells that transition to mesenchymal cells under inflammatory conditions [70]. CM from KO-PMF induced 2-fold higher chemotaxis that CM from WT-PMF, whereas the CM from WT- or KO-PC produced equally low levels of chemoattractants (Figure 4A, 4B).

We previously published that the loss of SSeCKS induced an Rb-dependent premature senescence in MEF, involving the loss of scaffolding by SSeCKS for both PKCα and δ [30]. Compared to WT-PMF, KO-PMF exhibited increased markers of cell senescence, such as p21 and p16 (Figure 4C), or senescence-associated β-galactosidase (Figure 4D). A notion posited recently is that cell senescence associated with the loss of tumor suppressor or the gain or oncogene functions may also lead to a senescence-associated secretory phenotype (SASP) [71] in which the increased secretion of chemokines and inflammatory mediators by mesenchymal cells helps promote metastasis by creating crosstalk between tumor and microenvironmental cells in the metastatic niche [72]. Indeed, KO-PMF secrete significantly higher levels of many chemokines involved in promoting inflammatory and metastatic responses (Figure 4E), arguing that the loss of SSeCKS in KO-PMF induces SASP. Moreover, KO-PMF upregulate several immune markers, such as F4/80 (*Adgre1*) and Cd11b (Figure 2G), suggesting that they gain markers normally found on fibrocytes, hematopoietic stem cell-derived fibroblast precursors involved in chronic inflammation [73].

To test whether KO-PMF compared to WT-PMF are more sensitized to secrete Cxcl10 by inflammatory mediators, equal numbers of cells grown in serum-free media were treated overnight with lipopolysaccharide (LPS), TNFα, IFNγ or IFNα and then the resulting media were tested for Cxcl10 levels by ELISA. KO-PMF secreted significantly higher Cxcl10 levels compared to WT-PMF in response to each of these mediators (Figure 4F). In contrast, neither IL-6, which is known to induce Cxcl10 in macrophages in a STAT3-dependent manner [74], nor GM-CSF, which is known to activate STAT5 but not Cxcl10 secretion [75], induced Cxcl10 secretion in KO- or WT-PMF over untreated background cells (Figure 4G). Pre-incubation with mouse Cxcl10-specific NAb abrogated the ability of the CM from IFNγ-treated KO-PMF, but not from WT-PMF, to induce enhanced chemotaxis of B16F10-luc cells (Figure 4H). It should be noted that PMF are not phenocopied by mouse embryo fibroblasts, as we showed that the latter (WT or KO) do not express Cxcl9 or 10 (data not shown), suggesting that Cxcl9/10 secretion is either specific to PMF or their environment. These data strongly suggest that the loss of SSeCKS hypersensitizes PMF to express and secrete Cxcl10 chemoattractant in response to inflammatory mediators typically produced by myeloid lineage cells, possibly those recruited in larger numbers to the peritoneum in KO mice.

**Figure 5:**
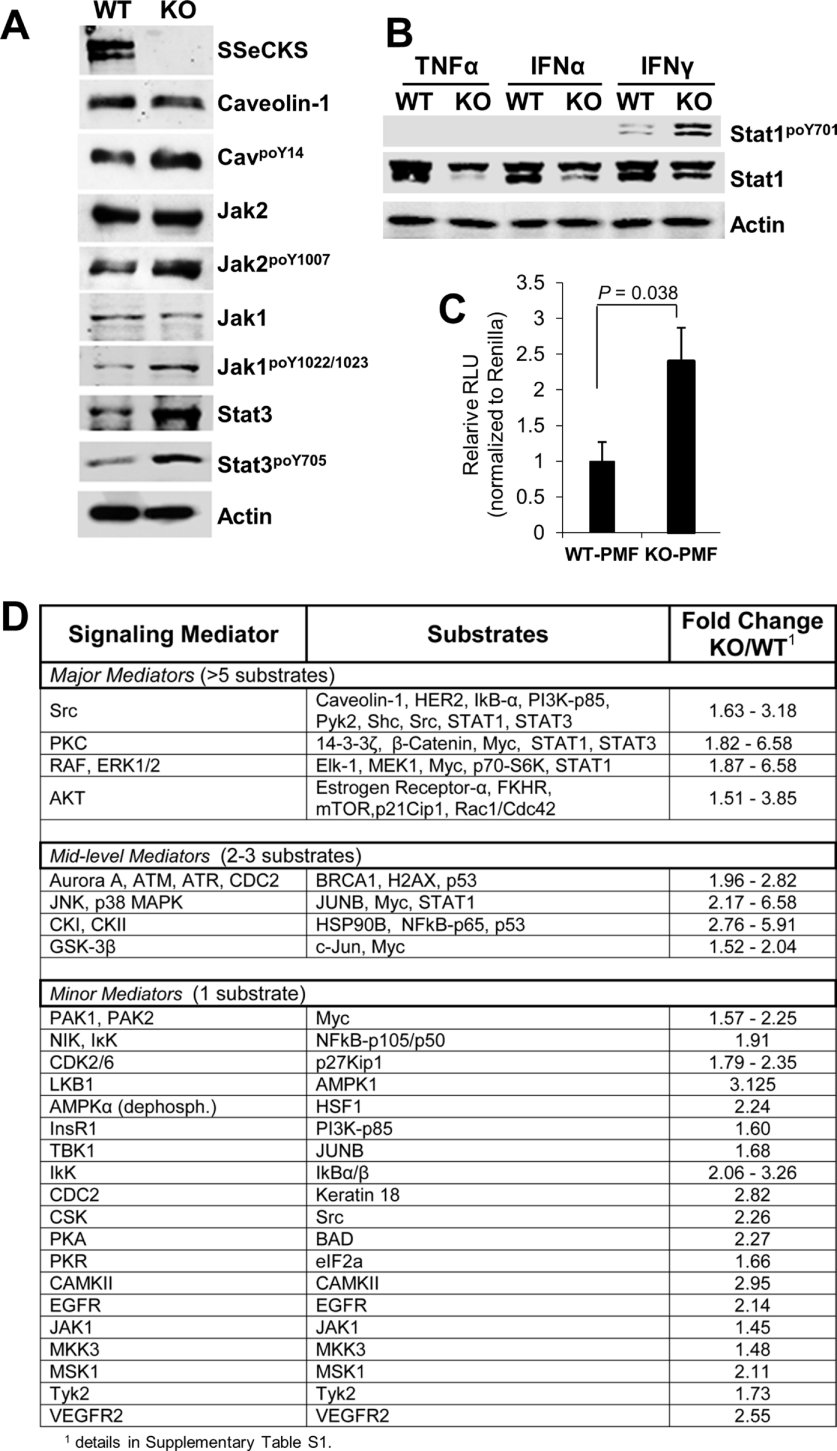
Signaling pathways activated in KO-PMF relative to those in WT-PMF (**A**) IB of IFNγ-treated (30 min) WT- or KO-PMF, probed for various total or phosphotyrosyl signaling mediators, or β-actin as a loading control. (**B**) IB of WT- or KO-PMF treated (30 min) with TNFα, IFNγ or IFNα, probed for STAT1^poY701^ vs. total STAT1 levels, or β-actin as a loading control. (**C**) Relative Light Units (RLU) of an NFκB-luciferase construct relative to a TK-renilla transfection control, as described previously [101], in transiently transfected WT- or KO-PMF. (**D**) Signaling mediators activated in IFNγ-treated KO- vs. WT-PMF, assessed using a signaling antibody array kit (Full Moon Biosystems). Relative signals (pixel counts) were quantified by the manufacturer and then normalized to internal controls as well to matched antibodies identifying total protein for a given mediator. The kinases responsible for increased substrate phosphorylation were identified by literature searches (Pubmed) and by Phosphosite (www.phosphosite.org) analysis, shown in Supplementary Table 1.

Several pathways are known to regulate the expression, translation and/or secretion of Cxcl10, including STAT1, NFkB, PKC and PI3K/AKT, depending on the cell type [63, 76–79]. To identify which pathways might be involved in the enhanced production of Cxcl10 in KO- vs. WT-PMF, we treated early-passage serum-starved PMF with IFNγ for 30 min, noting that levels are roughly twofold higher in KO- vs. WT-PF (Supplementary Figure 3A), and then assessed by immunoblot (IB) the relative activation levels of signaling mediators. IFNγ induced higher activation in KO-PMF of JAK1 and 2 (assessed by, respectively, poY^1007^ and poY^1022/1023^ signals relative to total protein levels) and STAT1 (assessed by relative poY^701^ levels), as well as increased relative phosphorylation of the Src substrate, caveolin-1^poY14^ (Figure 5A, 5B). IFNγ also induced total STAT3 and STAT3^poY705^ levels equally (Figure 5A). In contrast, neither TNFα nor IFNα induced STAT1 activation in the PMF (Figure 5B). Interestingly, IFNγ induced the expression of an NFκB-responsive reporter construct to a greater extent in KO- vs. WT-PMF (Figure 5C), strengthening the notion that KO-PMF have enhanced NFκB activation. Because SSeCKS is known to scaffold several major signaling mediators including PKC isoforms, Src, calmodulin, cyclins, phosphodiesterase, PKA [1] and Aurora A kinase [80], it is possible that the loss of SSeCKS leads to hyperactivation of several of these pathways. Moreover, the upregulation of STAT3 by IFNγ in the absence of SSeCKS is likely not relevant to Cxcl10 regulation because of the inability of IL-6 to enhance Cxcl10 expression in KO-PMF (Figure 4G).

To better identify pathways altered in KO-PMF that might regulate the enhanced Cxcl10 expression, we used an antibody array kit that assesses the relative activation of 20 signaling pathways using 1,358 antibodies specific for total vs. phosphorylated kinase substrates (Full Moon Biosystems, Cat. #SET100). Equal aliquots of protein lysate from serum-starved KO- or WT-PMF cells treated for 30 min with IFNγ were applied to antibody arrays, and after normalization to internal controls and identifying relative increases in substrate phosphorylation (normalized to total substrate protein) in KO- vs. WT-PMF (Supplementary Table 1), we identified three groups of mediators selectively activated in KO-PMF lysates (Figure 5D). The first, called “major mediators”, contain signaling mediators such as Src, PKC, RAF/ERK-1/2 or AKT based on the identification of > 5 substrates/pathway whose phosphorylation was enhanced ≥ 1.5-fold more in IFNγ-treated KO vs. WT PMF. “Mid-level mediators”, including Aurora-A/ATM/ATR/CDC2, JNK/p38MAPK, casein kinase-I/II or GSK-3β, identified 2–3 phospho-substrates/pathway relatively increased in KO cells. Finally, multiple potential, yet “minor mediators” identified only one such substrate per pathway or kinase, and these included many cases of autophosphorylation. The hyperactivation of Src and PKC signaling likely relates to the loss of specific SSeCKS scaffolding domains for these proteins, as we showed previously for epithelial cells and fibroblasts [30, 33–35]. The increased activation of RAF/ERK-1/2 is consistent with our previous data showing that SSeCKS attenuates serum- and adhesion-induced MEK/ERK-1/2 signaling [34–36, 81], again, likely through the loss of Src and PKC scaffolding by SSeCKS. Consistent with our earlier reports of activated AKT in SSeCKS*-*null prostates [29], we identified activated AKT signaling in our KO-PMF, although it remains unclear how this regulation is manifested by SSeCKS. Hyperactivation of Aurora-A/ATM/ATR/CDC2 signaling in the absence of SSeCKS is consistent with recent reports showing a role in G2/M control by human SSeCKS (Gravin) through the scaffolding of Polo-like kinase 1 and Aurora A [80, 82]. Importantly, the antibody array data are consistent with our immunoblotting data in Figure 4A, namely that KO-PMF lysates exhibit higher relative phosphorylation levels of Caveolin-1^Y14^ (a known Src phosphorylation site [83]) and JAK2^Y1007^ (a Src-dependent site [84]) relative to levels in WT-PMF.

The data above identify several possible hyperactive signaling pathways potentially responsible for the enhanced Cxcl10 expression in KO-PMF cells. We argued that the loss of specific SSeCKS scaffolding functions resulted in these hyperactivations, and thus, the reexpression of full-length SSeCKS should abrogate this in KO-PMF whereas expression of SSeCKS mutants lacking scaffolding domains for critical signaling mediators would phenocopy the SSeCKS-null condition. This assumes that the effect of SSeCKS scaffolding is either to directly suppress the activity of a signaling mediator, an example known in the case of PKC [33, 85, 86], or to attenuate its signaling by affecting subcellular compartmentalization, as in the case of Src [35].

**Figure 6:**
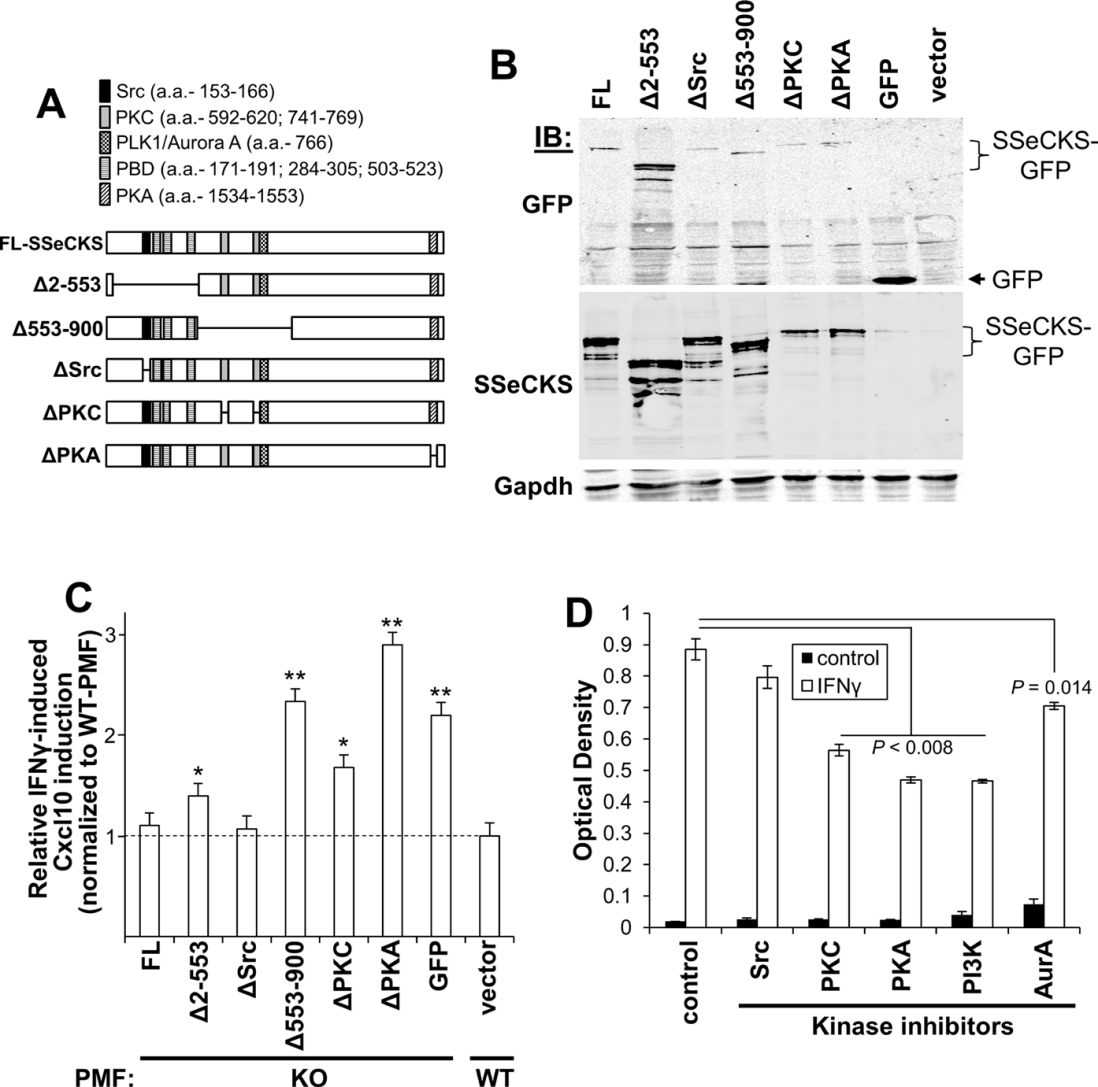
SSeCKS scaffolding activity controls Cxcl10 production via PKC, PKA and PI3K/AKT pathways (**A**) Full length (FL) and ΔSSeCKS mutants (GFP-fusions) relative to scaffolding domains known to bind signaling proteins or, in the case of the poly-basic domains (PBD), phosphoinositol phosphates (PIPs). (**B**) Comparable expression levels of the FL- and ΔSSeCKS proteins in KO-MEF assessed by IB for GFP or SSeCKS, with Gapdh as a loading control. (**C**) Relative IFNγ-induced Cxcl10 production in GFP-sorted KO-PMF transfected with FL or ΔSSeCKS mutants (vs. “GFP” vector control). IFNγ-treated WT-PMF were used as a normalization control for Cxcl10 levels. *p < 0.05; **p < 0.01. (**D**) Relative Cxcl10 levels (ELISA OD_560_) of KO-MEF first treated with various kinase inhibitors and then treated with PBS (control) vs. IFNγ for 30 min.

To identify which signaling mediators might be responsible, a panel of SSeCKS variants was produced as C-terminal GFP fusions containing either full-length (FL) protein or so-called “Δ” variants, containing deletions of discrete scaffolding regions. Specifically, ΔSrc, ΔPKC and ΔPKA contain 10-12 a.a. deletions that ablate binding to Src, PKC and PKA, whereas the Δ2-553 variant lacks binding sites for Src and phosphoinositol phosphates (PIPs bound by the polybasic binding domains [PBD]), and the Δ553-900 variant lacks dual binding domains for PKC and a scaffolding site for PLK1 and Aurora A (Figure 6A) [32, 33, 35, 80, 82, 85]. As a control to show that the various deletions did not induce protein instability, KO MEF were transfected with equal amounts of FL or ΔSSeCKS plasmid DNA and after two days, cell lysates were probed using GFP- or SSeCKS-specific antibodies, showing that the relative expression level of the SSeCKS protein panel was comparable (Figure 6B). To assess the effect of the SSeCKS variant panel on Cxcl10 secretion, early-passage WT and KO-PMF were transfected with SSeCKS-GFP constructs and after two days, GFP-positive cells were isolated by FACS, plated for one day, starved of serum overnight and then treated for 30 min with IFNγ or vehicle followed by Cxcl10-specific ELISA analysis. Paralleling the data in Figure 4F, IFNγ induced higher Cxcl10 expression in control (GFP-transduced) KO-PMF compared to WT-PMF (Figure 6C). Importantly, re-expression of FL SSeCKS abrogated the enhanced IFNγ-mediated induction of Cxcl10 in KO-PMF. The fact that ΔSrc also abrogated this effect suggests that the Cxcl10 induction is not controlled by Src signaling. In contrast, the reexpression of SSeCKS variants missing the scaffolding domains for PKC, PIPs, PLK1/Aurora A, or PKA maintained the enhanced IFNγ-mediated induction of Cxcl10 (Figure 6C).

To whether these pathways are required for the enhanced induction of Cxcl10 by IFNγ in KO-PMF, we pre-treated KO-PMF with inhibitors for Src (AZD0530) [87], PKC (*bis-*indolylmaleimide), PI3-kinase (LY294002), PKA (Rp-cAMPS) or Aurora kinase A (VX680) or vehicle followed by IFNγ for 30 min and Cxcl10 -specific ELISA analysis. Figure 6D shows that PKC, PKA and PI3K/AKT likely play critical roles in Cxcl10 production by KO-PMF whereas Src signaling was not a factor, agreeing with the results in Figure 6C, and Aurora A kinase played a small, though significant role. Taken together, these data strongly suggest that hyperactive PKC, PKA and PI3K/AKT pathways, occurring because of the loss of SSeCKS scaffolding functions, induce the enhanced levels of Cxcl10 by PMF that drive the increased metastatic chemotaxis of B16F10 cells to the peritoneum.

## DISCUSSION

There is growing appreciation for the role played by TME cells in the development of the pre-metastatic niche, such as after interaction with and education by tumor-secreted exosomes [88], and upon tumor cells colonization, in the cross-talk that either promotes or suppresses metastasis formation [16]. Having shown in multiple systems that SSeCKS/AKAP12 loss in tumor cells promotes metastasis in xenograft and transgenic models, and that this correlates with more SSeCKS/AKAP12 downregulation in human metastases compared with primary-site tumors [1], we addressed whether the documented loss of SSeCKS/AKAP12 in TME also facilitates increased metastatic potential. The current study presents evidence that SSeCKS/AKAP12 suppresses metastasis in the peritoneum by modulating key signaling mediators that control secretion of tumor chemoattractants. In the case of B16F10 melanoma cells, the chemoattractants produced by PMF are the Cxcr3 ligands, Cxcl9 and 10, and most likely, SSeCKS regulates production of Cxcl9/10 in PMF through its control of PKC, PKA and PI3K/AKT signaling.

Our data indicate that, compared to WT C57BL/6 controls, *Akap12-*null mice suffer from an increased burden of macrometastases in multiple organs, derived from spontaneous metastasis of *s.c.* tumors and from experimental metastases induced by *i.v.* tumor injections. Though the peritoneum is not a major *primary* site of melanoma metastasis described in clinical cases, almost half of cases identified because of metastases to other sites also exhibited peritoneal lesions [57]. Indeed, enhanced peritoneal metastasis occurred notwithstanding of *Braf* activation since both B16F10 (WT-*Braf*) and SM1WT1-LM3 (*Braf*^V600E^) induced higher levels on KO compared to WT hosts. We showed that the enhanced peritoneal metastasis of B16F10 in KO mice could be transferred by cell-free PF but not by PC, even though KO-PC contained more B-cells, fewer dendritic cells and more immature monocytes/macrophages than WT-PC (with no increases in total cells/ml). Cxcl9/10 are known chemoattractants for subsets of activated/memory T cells [89], and although there is no significant difference in total Cd3^+^ cells in WT vs. KO PC populations, we cannot exclude that KO PCs have increased subsets of activated/memory T cells or that these subsets increase more in KO PCs in response to tumor-secreted inflammatory mediators.

The notion that KO-PF contained higher levels of melanoma chemoattractants was confirmed using chemotaxis assays as well as our demonstrating increased levels of many inflammatory mediators, interleukins and chemokines using antibody arrays. That the increased levels of Cxcl9 and 10 in KO-PF were critical for the enhanced peritoneal metastasis was confirmed by data showing that knockdown of Cxcr3 in B16F10 and SM1WT1-LM3 abrogated enhanced metastasis *in vivo* and/or enhanced chemotaxis *in vitro.* We then showed that KO-PMF, but not KO-PC, exhibited higher levels of spontaneous and IFNγ-induced Cxcl10 secretion. Interestingly, stromal cells induce Cxcl10 under conditions of inflammation and senescence, and this correlates with our previous results showing that KO-MEF suffer from Rb-dependent premature senescence [30], resulting in the upregulation of VEGF, IL6 and other factors describing a SASP condition [90]. SSeCKS is known to be normally upregulated by inflammatory mediators such as LPS or TNFα [91, 92], and thus, SSeCKS downregulation in tumor-associated stroma must result from tumor-derived cross-talk factors. It could be argued, therefore, that loss of SSeCKS in PMF causes a local inflammatory/senescence environment that tumor cells sense as a pre-metastatic niche. Indeed, many groups have now endeavored to therapeutically target such TME niches as a means of preventing or ameliorating metastatic progression [90].

The notion that SSeCKS controls cellular senescence *in vivo* is backed by data showing that some tissues normally enriched for SSeCKS expression are mildly hyperplastic in KO mice, including the prostate [29], skin [27] and kidney mesangial cells [93]. Moreover, these hyperplastic tissues express higher levels of senescence markers, such as SA-β-gal, p16^ink4a^, p21 and γ-H2AX. In addition, the loss of SSeCKS accentuates injury-induced dysfunction to barrier functions in the kidney [93] and brain [94], suggesting that SSeCKS protects against stress-induced senescence. Histologic analyses of WT and KO peritoneal membranes (from which the PMF were derived) showed otherwise normal structures, yet the KO membranes had roughly 1.8-fold more PMF than those from WT mice, based on staining for α-smooth muscle actin (data not shown). Importantly, KO-PMF exhibited higher levels of the senescence markers, SA-β-gal, p16^ink4a^ and p21. Thus, based on our current data showing increased secretion of chemokines such as Cxcl10 by KO-PMF, it is likely that these cells are suffering from increased senescence.

The importance of tumor-encoded Cxcr3 in promoting metastatic chemotaxis and cancer progression has been well documented [42]. Increased tumor Cxcr3 expression has been linked to increased incidence of metastasis and poorer patient prognosis in several cancer types [41], including melanoma [47], especially those cases expressing WT-BRAF [95]. Kawada et al. [44] showed that Cxcr3 plays a role B16F10 metastasis to lymph nodes, and although this study did not clarify whether this was due to increased chemotaxis ability, Dengel et al. [96] showed that treatment of human LN samples with IFNγ could induce human melanoma chemoattraction *in vitro* through the upregulated expression of CXCL9/10/11. Interestingly, Cambien et al. [49] used human and mouse colon cancer models to show that systemic treatment with a small molecule CXCR3 antagonist, AMG487, could suppress metastasis in an organ-specific manner. Our current study, however, is the first to describe a mechanism that controls Cxcr3 ligand secretion in TME cells by a metastasis suppressor, and moreover, it is the first to address how IFNγ-induced Cxcr3 ligand expression by PMF facilitates melanoma metastasis to the peritoneum. Importantly, knockdown of Cxcr3 in our B16F10-luc system only affected metastasis and did not affect primary tumor growth.

Our genetic (using SSeCKS scaffolding domain mutants) and signaling inhibitor data suggest that hyperactivated PKC, PKA and PI3K/AKT, but not Src or Aurora A kinase, are responsible for enhanced production of Cxcl10 in KO-PMF. Although it is unclear how SSeCKS attenuates PI3K/AKT signaling, we assume that it is affected by the ability of SSeCKS to scaffold PIPs, based on the finding that the Δ2-553 mutant only partially diminishes IFNγ-induced Cxcl10 production in KO-PMF. As noted, PKC is known to play a role on IFNγ-induced Cxcl10 expression [79], but this regulation, as well as the ability of PKA to regulate this pathway, is much understudied.

In sum, the current study describes a mechanism by which SSeCKS attenuates peritoneal metastasis of experimental melanoma models by controlling the expression and secretion of Cxcl9/10 by specific peritoneal TME cells, PMF, in PKC-, PKA- and PI3K/AKT-dependent manners. These data suggest that in addition to suppressing metastasis through tumor-specific mechanisms, SSeCKS can also attenuate metastasis at specific “soil” sites by controlling the secretion of tumor chemoattractants.

## MATERIALS AND METHODS

### Cell line and culture

The B16-F10-luc-G5 (“B16F10-luc”) murine melanoma cell line was purchased from Perkin Elmer/Caliper Life Sciences (Akron, OH) and maintained in RPMI1640 supplemented with 10% fetal bovine serum (FBS) and grown at 37°C in 5% CO_2_. SM1WT1 cells were a gift of Dr. Liam Town (Peter MacCallum Cancer Institute). After transducing with pLenti-CMV-PURO-Luc Addgene plasmid #17477; deposited by Eric Campeau) and selecting for puromycin-resistant growth, cells (10^6^/mouse) were injected *i.v.* tail-vein*)* into male C57BL/6J mice, and after 4 weeks, macrometastases harvested from lungs. The cells were cultured in RPMI1640 plus 10% FBS, and subjected to two more rounds of *in vivo* selection for lung metastases, resulting in SM1WT1-LM3-luc cells. HeLa cells were maintained in DMEM/10% FBS. Peritoneal membrane fibroblasts (PMF) dissected under sterilize conditions from neonatal peritoneal membranes were minced into < 1 mm fragments and digested with Dispase and DNase I for 1hr at 37°C, then filtered through a sterile 70 µm mesh (Corning-Falcon, Corning, NY). Immortalized mouse embryonic fibroblast (MEF) [30] or PMF were cultured in DMEM/10% FBS containing non-essential amino acids, L-glutamine, 2-mercaptoethanol, sodium pyruvate, HEPES and penicillin-streptomycin.

### Plasmid construction

Construction of SSeCKS-GFP in pcDNA3.1 was described previously [97]. The following deletions were produced in pcDNA3.1/SSeCKS-GFP using long-run inverse PCR, as we described previously [98]: ΔSrc (a.a. 153–166) [35], ΔPKC (a.a. 596-605, 745–753) [33], ΔPKA (a.a. 1534–1553) (this study), Δ553–900 [33] and Δ2-553 [97].

### Animal studies

All mouse care and experiments were performed in accordance with established institutional guidelines and approval by the Roswell Park Cancer Institute Animal Care and Use Committee. SSeCKS/*Akap12*-null (KO) mice were generated as described previously [29]. C57BL/6J wild-type (WT) mice were obtained from Jackson Laboratory (Bar Harbor, ME). Mice injected with cancer cell lines were 5 to 7-week-old mice and primary PMF cells were obtained from 1 to 2 week-old mice.

For *in vivo* proliferation assays, 2 × 10^5^ B16F10-luc cells were injected *s.c.* in the flank of 6 week-old WT or KO mice. Primary tumors were grown for 21d, during which tumor volumes (mm^3^) were determined by the formula, 0.52 × L × W × H.

For the adoptive transfer of peritoneal fluid (PF) or cells (PC), fluid was collected by injecting 3 ml of sterilized PBS into the peritoneal cavity of anesthetized mice, and after massaging the midsection briefly, the fluid was slowly aspirated and collected, then separated into PF and PC following centrifugation at 100g for 10 min at room temperature. 1 ml of PF or 10^6^ PC were injected *i.p.* into naïve mice, which were then challenged 24 h later with 2 × 10^5^ B16F10-luc tumor cells (*i.v.* or *s.c.*). Macrometastases were identified as melanin-rich brown lesions.

### Cell migration and motility assays

For chemotaxis assays, serum-free DMEM (SFM) supplemented with 10% PF from WT or KO mice or 20% conditioned media (CM) from WT- or KO-PMF was placed into 24 well culture plates; 5 × 10^4^ B16F10-luc[shCxcr3] or [shCntrl] cells suspended in SFM and applied atop 8.0 µm Boyden chamber inserts (BD Falcon). In some cases, 1 μg of mouse Cxcl10 neutralizing Ab (NAb)(#AF-466, R&D Systems, Minneapolis, MN) or control goat Ig (#G9023, Sigma-Aldrich, St. Louis, MO) were pre-incubated with bottom media for 1h at RT. After 16h the inserts were removed and the non-migrating cells wiped from atop inserts, followed by staining (with Diff-Quick; Siemens, Erlangen, Germany) and counting of the migrated cells (at bottom of the insert membrane). For wound healing assays, B16F10-luc[shCxcr3] or [shCntrl] monolayer cell cultures in 6-well plates were scratched and migrated cells quantified after 12h as performed previously [99].

### *In vitro* proliferation assays

B16F10 tumor cells were seeded 2 × 10^3^ cells/well in 96-well culture plates. After 2, 24, 48, 72 and 96 h, the medium was removed and the cells fixed with cold methanol for 10 min. The cells were then treated with 100 µl of 0.5% crystal violet solution (50 mg crystal violet in 25% methanol) 10 min, followed by extensive washing with water and complete drying. Stained cells were dissolved for 20 min in 100 µl of 10% acetic acid, and then absorption was measured at 595 nm using a microplate reader (SpectraMax-M2, Molecular Devices, (Sunnyvale, CA).

### SA-βgal staining

Staining was performed as described previously [30].

### Immunoblotting (IB)

Cells were lysed in RIPA buffer [100] containing 1 mM each of phenylmethanesulfonylfluoride, Na_3_VO_4_, NaF plus one Complete Protease Inhibitor (Roche, Indianapolis, IN) tablet per 10 ml. Lysates were cleared by centrifugation at 15,000 × g for 10 min after sonication. The following antibodies were used: CXCR3 (1:500, sc-13951, Santa Cruz, Santa Cruz, CA), p16 (1:500, sc-1661, Santa Cruz), p21 (1:500, sc-397, Santa Cruz), Stat1 (1:1000, sc-346, Santa Cruz), Stat3 (1:1000, sc-482, Santa Cruz), Stat5 (1:1000, #9363, Cell Signaling, Danvers, MA), po-Stat1 (1:1000, #9171, Cell Signaling), po-Stat3 (1:1000, sc-8059, Santa Cruz), po-Stat5 (1:1000, #9351, Cell Signaling) and Gapdh (1:1000, sc-25778, Santa Cruz). For the analysis of soluble protein expression profiles of the PF, we used the Proteome Profiler Array mouse chemokine array kit (R&D Systems) using 200 µl of WT- or KO-PF. The intensities of the immunoreactive IB bands and array spots were quantified by densitometry using ImageJ software (NIH).

### RT-PCR and quantitative real-time PCR

Total RNA fraction was extracted from cells using the TRIZOL reagent (ThermoFisher-Invitrogen, Grand Island, NY), and was reverse transcribed using Superscript III reverse transcriptase (Invitrogen) for RT-PCR and High capacity cDNA reverse transcription kit (ThermoFisher-Applied Biosystems, Grand Island, NY) for quantitative real-time PCR. Quantitative real-time PCR was performed on a 7900HT real-time PCR system (Applied Biosystems) using SYBR Green PCR Core Reagents (Applied Biosystems). The relative expression was normalized to β-actin.

### Lenti/retrovirus packaging

Lentiviruses were produced by co-transfecting HEK-293T cells (ATCC: CRL-11268) with pHIV-dTomato (Addgene #21374) or pGIPZ (GE-Dharmacon/OpenBiosystems) vectors plus the packaging constructs pCMVdeltaR8.2 and pMD2.G (gifts of the D. Trono lab, Basal Switzerland). Phoenix ectopic packaging cells (ATCC: CRL-3214) were transfected with pSM2 retrovirus plasmids in LipoD293 and incubated at 32°C for 72 h. The mouse *Cxcr3* shRNA sense sequences used were V2MM_62973: 5′-ACCCATCTCAGTATCTCAATAT-3′ (clone-1) and V2MM_67318: 5′-AGCCTCCTACCTGGGCTTGTAA-3′ (clone-2) (Roswell Park Cancer Institute shRNA Core Resource, Irwin Gelman, Ph.D., Director).

### Cxcl10 ELISA

Cells grown to 80% confluency in 12-well dishes were starved overnight in media with 0.5% FBS, then treated overnight with AZD0530 (100 nM, LC Laboratories, Wobum, MA), *bis*-indolylmaleimide (500 nM; R&D Systems), LY294002 (50 μM, # 1130, R&D Systems), Rp-cAMPS (100 nM, sc-24010, Santa Cruz), VX680 (20 nM; gift of T. Ouchi, Roswell Park Cancer Institute), or PBS control, with or without treatment with IFNγ (100 ng/ml, R&D Systems). Media samples were then centrifuged at 2000 RPM for 5 min, and 100 μl taken for mouse-specific CXCL10-ELISA analysis (#DY466, R&D Systems). All experiments were done in triplicate, and repeated at least twice.

### Antibody array

To investigate the activated intracellular signal pathways by loss of SSeCKS, antibody array (#SET100, Full Moon BioSystems, Sunnyvale, CA) was performed following a protocol provided by manufacturer. Briefly, cellular proteins were extracted from 5 × 10^6^ cells with lysis buffer and lysis beads, and purified by column provided with the kit. Purified proteins were quantified and biotinylated, and coupled with antibody array platform. After conjugation with Cy3-streptavidin and washing with PBS, the antibody array slides were scanned and quantified by manufacturer.

### Statistical analyses

Data were expressed as mean ± SEM, with all experiments repeated independently at least twice. Differences were analyzed by one-way ANOVA or Student’s *t* test. *P* < 0.05 was considered significant.

## SUPPLEMENTARY MATERIALS FIGURES AND TABLE





## Abbreviations

a.a: amino acids
AKAP12: A kinase anchoring protein 12
ANOVA: Analysis of variance
CM: conditioned media
CSF-1: colony stimulating factor-1
EGF: epidermal growth factor
ELISA: enzyme-linked immunosorbent assay
FBS: fetal bovine serum
FL: full length
GFP: green fluorescent protein
IFN: interferon
*i.p*: intraperitoneal
*i.v*: intravenous
KO: knockout
LM3: lung metastasis 3
Luc: luciferase
MEF: mouse embryo fibroblast
MN: metastatic niche
NS: not significant
PBD: poly-basic domain
PC: peritoneal cells
PF: peritoneal fluid
PI3K: phosphoinositol-3-kinase
PIP: phosphoinositol phosphates
PKA: protein kinase A
PKC: protein kinase C
PMF: peritoneal myofibroblast
SASP: senescence associated secretory phenomenon
*s.c*: subcutaneous
SDF-1: stromal cell-derived factor 1
SEM: standard error of the mean
SFK: Src-family kinases
SSeCKS: Src Suppressed C Kinase Substrate
RT-PCR: reverse transcriptase-polymerase chain reaction
TME: tumor microenvironment
TNF: tumor necrosis factor
VEGF: vascular endothelial growth factor
WT: wild-type
