# Supplementary material for: SSeCKS/AKAP12 scaffolding functions suppress B16F10-induced peritoneal metastasis by attenuating CXCL9/10 secretion by resident fibroblasts
Source: Oncotarget. 2017 Aug 9;8(41):70281–98. doi: 10.18632/oncotarget.20092 (PMC5642554; doi:10.18632/oncotarget.20092)
Supplement: Supplementary file 1 [file oncotarget-08-70281-s001.pdf]

SSeCKS/Akap12 scaffolding functions suppress B16F10-induced peritoneal metastasis by attenuating Cxcl9/10 secretion by resident fibroblasts

SUPPLEMENTARY MATERIALS

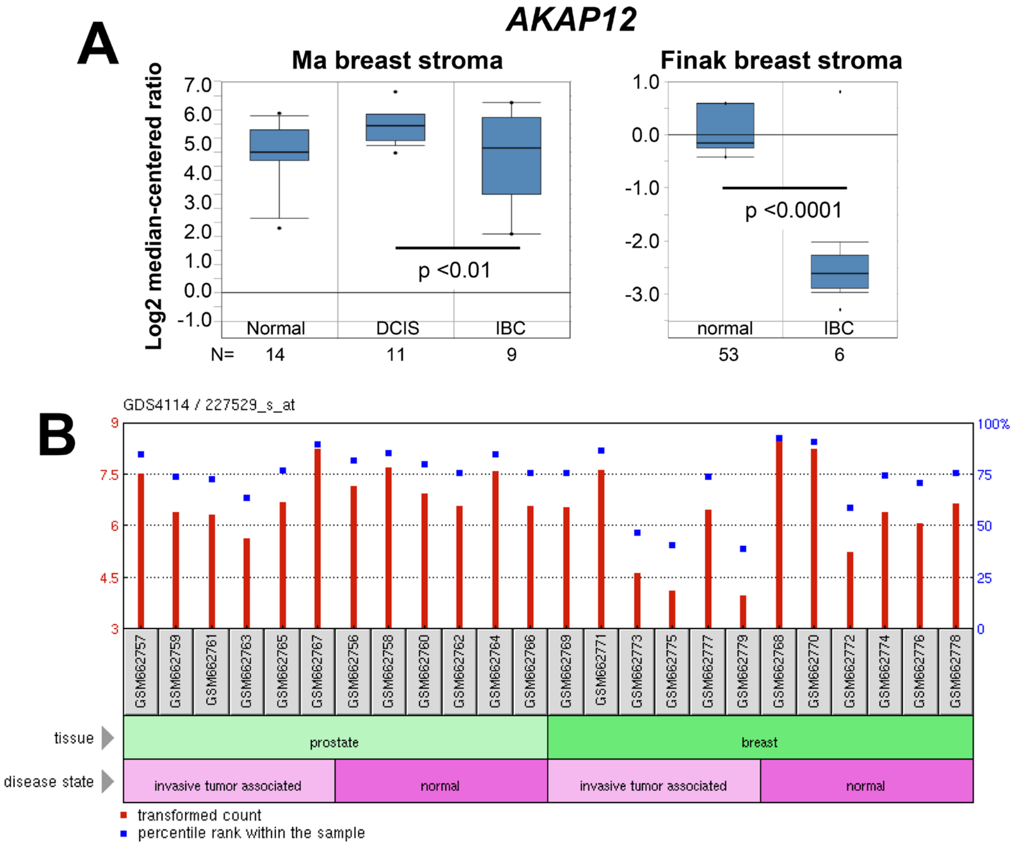

Supplementary Figure 1: (**A**) Downregulation of SSeCKS/*AKAP12* in the tumor-associated stroma in breast (normal vs. DCIS, ductal carcinoma *in situ* vs. IBC, invasive breast cancer) and/or prostate (cancer vs. normal) from Oncomine studies (Oncomine.org) of Ma and Finak (Gene Expression Omnibus studies GSE14548 and GSE12622, respectively). (**B**) Gene Expression Omnibus study GDS4114 of Planche comparing reactive stroma of breast or prostate cancer to normal stroma. Stroma were isolated using laser-capture microdissection.

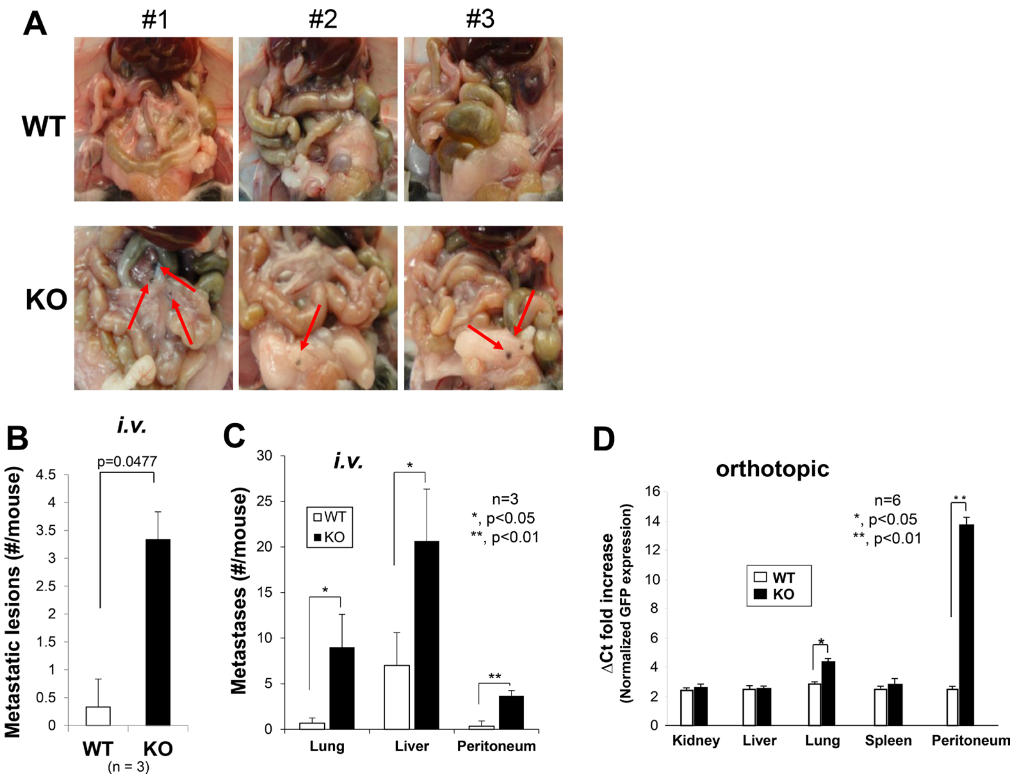

Supplementary Figure 2: Increased peritoneal metastasis in KO mice. (**A**) Examples of peritoneal macrometastases (red arrows). (**B**) Average number of metastases/mouse in WT vs. KO hosts (n = 3) derived from *Akap12*$^{+/-}$ mice, injected i.v. with B16F10-luc. (**C**) Average number of metastases/mouse in the liver, lung and peritoneum of WT or KO mice (n = 3) injected i.v. with B16F10-luc cells. (**D**) Quantification of metastatic burden from orthotopic tumors of GFP-labeled B16F10-luc cells, based on isolation of organ DNA 4 weeks after injection followed by qPCR for GFP (F: 5'-AAGCTGACCCTGAAGTTCATCTGC-3', R: 5'-CTTGTAGTTGCCGTCGTCCTTGAA-3'). Error bars, S.E. of triplicate assays of DNAs pooled from 6 mice each.

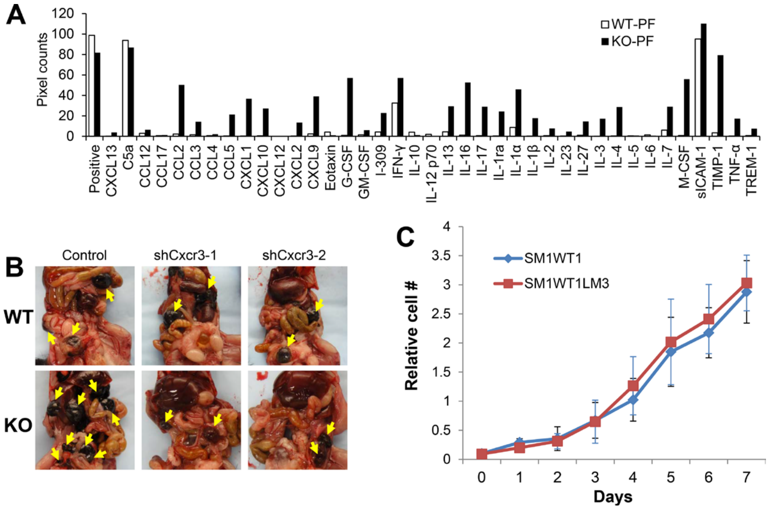

Supplementary Figure 3: (**A**) Pixel counts from Proteome Profiler assay described in Figure 2A using WT- or KO-PF pooled from 6 mice each. (**B**) Peritoneal metastases (yellow arrows) from WT or KO mice injected *i.v.* with B16F10-luc[EV] ("control"), B16F10-luc[shCxcr3-1] or B16F10-luc[shCxcr3-2]. (**C**) Proliferation (relative cell numbers) of SM1WT1-luc vs. SM1WT1-LM3-luc cells.

Supplementary Table 1: Signaling antibody array data comparing fold changes in WT vs. KO PMF, and known kinasespecific substrate phosphorylations. See Supplementary_Table_1
