# Supplementary material for: SSeCKS/AKAP12 scaffolding functions suppress B16F10-induced peritoneal metastasis by attenuating CXCL9/10 secretion by resident fibroblasts
Source: Oncotarget. 2017 Aug 9;8(41):70281–98. doi: 10.18632/oncotarget.20092 (PMC5642554; doi:10.18632/oncotarget.20092)
Supplement: Supplementary file 2 [file oncotarget-08-70281-s002.docx]

| **Supplementary Table 1** | | |
| --- | --- | --- |
| **Protein List** | **Fold Change** | **Substrate Site** |
|  | **WT/KO** |  |
| 14-3-3 ζ (Ab-58) | 1.61 |  |
| 14-3-3 ζ (poS58) | 0.52 | PKCδ |
| AMPK1 (Ab-174) | 1.42 |  |
| AMPK1 (poT174) | 0.46 | LKB1 |
| BAD (Ab-112) | 1.06 |  |
| BAD (Ab-136) | 1.19 |  |
| BAD (Ab-155) | 1.25 |  |
| BAD (poS155) | 0.51 | PKA |
| β-Actin | 1.71 |  |
| β-Catenin (Ab-37) | 1.77 |  |
| β-Catenin (Ab-41/45) | 1.55 |  |
| β-Catenin (poS33) | 0.91 | phos by PKCα, leads to β-Catenin degradation |
| β-Catenin (poS37) | 0.28 |  |
| β-Catenin (poT41/S45) | 0.55 |  |
| BRCA1 (Ab-1423) | 1.33 |  |
| BRCA1 (Ab-1524) | **1.34** |  |
| BRCA1 (poS1524) | 0.65 | Aurora A |
| CaMKII (Ab-286) | 1.77 |  |
| CaMKII (poT286) | 0.60 | autophos site |
| Caveolin-1 (Ab-14) | 1.62 |  |
| Caveolin-1 (poY14) | 0.51 | Src |
| c-Jun (Ab-243) | 1.00 |  |
| c-Jun (Ab-73) | 1.09 |  |
| c-Jun (poS243) | 0.69 | GSK-3β |
| EGFR (Ab-1110) | 1.48 |  |
| EGFR (poY1110) | 0.69 | autophos |
| eIF2a (Ab-51) | 1.16 |  |
| eIF2a (poS51) | 0.70 | PKR |
| Elk-1 (Ab-383) | 0.99 |  |
| Elk-1 (poS383) | **0.53** | ERK |
| Estrogen Receptor-α (Ab-167) | 1.27 |  |
| Estrogen Receptor-α (poS167) | 0.66 | AKT |
| FKHR(Ab-256) | 0.96 |  |
| FKHR(poS256) | **0.49** | AKT |
| GAPDH | 1.23 |  |
| HER2 (Ab-877) | 1.18 |  |
| HER2 (poY877) | 0.61 | Auto- or Src |
| Histone H2A.X (Ab-139) | 1.92 |  |
| Histone H2A.X (poS139) | 0.98 |  |
| HSF1 (Ab-303) | **1.12** |  |
| HSF1 (poS303) | 0.50 | dephos AMPKa |
| HSP90B (Ab-254) | 1.30 |  |
| HSP90B (poS254) | 0.22 | CKII |
| IkB-α (Ab-32/36) | 1.00 |  |
| IkB-α (Ab-42) | 1.35 |  |
| IkB-α (poS32/poS36) | 1.31 |  |
| IkB-α (poY42) | 0.57 | Src |
| IkB-β (poS23) | 0.36 | IkK |
| IkB-ε (Ab-22) | 1.38 |  |
| IkB-ε (poS22) | 0.46 | IkK |
| JAK1 (Ab-1022) | 1.20 |  |
| JAK1 (poY1022) | 0.83 |  |
| JAK2 (Ab-1007) | 1.26 |  |
| JAK2 (Ab-221) | 0.97 |  |
| JAK2 (poY1007) | 0.74 | Abl |
| JAK2 (poY221) | 0.80 |  |
| JunB (Ab-259) | 1.37 |  |
| JunB (Ab-79) | 1.41 |  |
| JunB (poS259) | 0.83 |  |
| JunB (poS79) | 0.64 | JNK |
| JunD (Ab-255) | 1.31 |  |
| JunD (poS255) | 0.79 |  |
| Keratin 18 (Ab-33) | 1.07 |  |
| Keratin 18 (poS33) | 0.38 | cdc2 |
| MEK1 (Ab-217) | 1.41 |  |
| MEK1 (Ab-221) | 1.54 |  |
| MEK1 (Ab-291) | 1.18 |  |
| MEK1 (poS217) | 0.36 | RAF |
| MKK3 (Ab-189) | 1.30 |  |
| MKK3 (poS189) | 0.88 |  |
| MSK1 (Ab-376) | 1.29 |  |
| MSK1 (poS376) | 0.61 |  |
| mTOR (Ab-2448) | 1.09 |  |
| mTOR (poS2448) | 0.72 | Akt1 |
| Myc (Ab-358) | 1.34 |  |
| Myc (Ab-373) | 1.56 |  |
| Myc (Ab-58) | 1.45 |  |
| Myc (Ab-62) | **1.94** |  |
| Myc (poS373) | **0.70** | PAK2 |
| Myc (poS62) | 0.51 | ERK, JNK |
| Myc (poT358) | 1.00 |  |
| Myc (poT58) | 0.77 | ERK2, GSK3b, PKCε |
| NFkB-p105/p50 (Ab-337) | **1.08** |  |
| NFkB-p105/p50 (Ab-893) | 1.05 |  |
| NFkB-p105/p50 (Ab-907) | 1.07 |  |
| NFkB-p105/p50 (poS893) | 0.56 | NIK & IkKa |
| NFkB-p105/p50 (poS907) | 0.87 |  |
| NFkB-p105/p50 (poS932) | 1.10 |  |
| NFkB-p65 (Ab-254) | **1.19** |  |
| NFkB-p65 (Ab-529) | 1.30 |  |
| NFkB-p65 (poS529) | 0.63 | CKII |
| NFkB-p65 (poT254) | 0.71 |  |
| p21Cip1 (Ab-145) | 1.31 |  |
| p21Cip1 (poT145) | **0.78** |  |
| p27Kip1 (Ab-10) | 1.32 |  |
| p27Kip1 (Ab-187) | 1.22 |  |
| p27Kip1 (poS10) | 0.71 | CDK6 |
| p27Kip1 (poT187) | **0.54** | CDK2 |
| P38 MAPK (Ab-182) | 1.30 |  |
| P38 MAPK (poT180) | 1.24 |  |
| P38 MAPK (poY182) | 1.07 |  |
| p44/42 MAPK (Ab-202) | 1.09 |  |
| p44/42 MAPK (Ab-204) | 1.45 |  |
| p44/42 MAPK (poT202) | 1.38 |  |
| p44/42 MAPK (poY204) | 1.26 |  |
| p53 (Ab-315) | 1.37 |  |
| p53 (Ab-6) | 1.17 |  |
| p53 (poS315) | 0.55 | Aurora A |
| p53 (poS6) | 0.46 | CK1δ and CK1ε |
| p70 S6 Kinase (Ab-424) | 1.46 |  |
| p70 S6 Kinase (poS424) | 0.38 | ERK and PI3K |
| PI3-kinase p85-α (Ab-607) | 1.36 |  |
| PI3-kinase p85-α (poY607) | 0.85 | InsulinR |
| PI3-kinase p85-α/γ (Ab-467/199) | 1.24 |  |
| PI3-kinase p85-α/γ (poY467/poY199) | 0.76 | Src |
| Pyk2 (Ab-402) | 1.37 |  |
| Pyk2 (poY402) | **0.69** | autophos & Src |
| Rac1/cdc42 (Ab-71) | 1.54 |  |
| Rac1/cdc42 (poS71) | 0.40 | AKT |
| Rel (Ab-503) | 1.18 |  |
| Rel (poS503) | 0.62 |  |
| Shc (Ab-349) | 1.81 |  |
| Shc (poY349) | 0.53 | Src |
| Src (Ab-418) | 1.37 |  |
| Src (Ab-529) | 1.42 |  |
| Src (poY418) | 0.84 | autophos |
| Src (poY529) | 0.62 | Csk |
| STAT1 (Ab-701) | 1.72 |  |
| STAT1 (Ab-727) | 1.43 |  |
| STAT1 (poS727) | 0.24 | p38 MAPK |
| STAT1 (poY701) | 0.93 |  |
| STAT3 (Ab-705) | 1.51 |  |
| STAT3 (Ab-727) | 1.54 |  |
| STAT3 (poS727) | 0.74 | PKCδ/ε |
| STAT3 (poY705) | 0.85 | Src |
| TYK2 (Ab-1054) | 1.30 |  |
| TYK2 (poY1054) | 0.75 | IFNαR1 |
| VEGFR2 (Ab-951) | 1.49 |  |
| VEGFR2 (poY951) | 0.42 | autophosp |
